# Ornithine decarboxylase antizyme 2 (OAZ2) in human colon adenocarcinoma: a potent prognostic factor associated with immunity

**DOI:** 10.1038/s41598-025-90066-4

**Published:** 2025-03-03

**Authors:** Yiheng Liu, Shengjie Zhang, Wenjie Liao, Jun Qian, Cuihua Lu, Li Jin

**Affiliations:** 1 Department of Gastroenterology, Affiliated Hospital of Nantong University, Medical School of Nantong University, Nantong, Jiangsu China; 2 Department of Emergency, Nantong Third People’S Hospital, Affiliated Nantong Hospital 3 of Nantong University, No. 60, Qingnian Middle Road, Chongchuan District, Nantong, 226000 Jiangsu People’s Republic of China; 3 Department of Emergency, Lianyungang Second People’S Hospital, Affiliated to Kangda College of Nanjing Medical University, Lianyungang, Jiangsu China; 4 Department of Gastroenterology, Affiliated Hospital of Nantong University, Nantong, , Jiangsu China; 5 Department of Emergency Medicine, the First Affiliated Hospital of Soochow University, Suzhou, China

**Keywords:** OAZ2, COAD, Prognosis, Immunity, Overall survival, Biochemistry, Cancer, Cell biology, Immunology, Molecular biology, Biomarkers, Oncology

## Abstract

Despite few studies focusing on the OAZ2 gene in colorectal cancer, its potential role in colon adenocarcinoma (COAD) prognosis and immune modulation remains underexplored. This study examines the expression and mechanistic involvement of OAZ2 in COAD using data from The Cancer Genome Atlas (TCGA) and additional laboratory experiments. We employed uni- and multivariate Cox hazard regression analyses to evaluate its prognostic significance and gene set enrichment analysis (GSEA) to identify related signaling pathways. Our findings demonstrate significantly lower OAZ2 expression in COAD tissues compared to normal counterparts (P < 0.05) and establish its value as an independent prognostic indicator (P < 0.05). Laboratory experiments further revealed that the protein and mRNA levels of OAZ2 are significantly diminished in COAD compared to adjacent normal tissues, while its antagonist AZIN2 shows elevated expression, suggesting a competitive interaction that may regulate tumor behavior. Overexpression of OAZ2 in RKO colorectal cancer cells significantly reduced their proliferation rate and impaired migration, confirming the functional impact of OAZ2 dysregulation in COAD. Gene Set Enrichment Analysis (GSEA) highlighted the involvement of OAZ2 in cardiac muscle contraction and oxidative phosphorylation pathways. Additionally, OAZ2’s association with immune features such as tumor mutational burden (TMB), microsatellite instability (MSI), and immune infiltration underscores its integral role in the tumor microenvironment. These comprehensive findings position OAZ2 as a promising biomarker for COAD prognosis and a potential target for therapeutic intervention, with evidence supporting its regulatory effects on cell dynamics and tumor aggressiveness.

## Introduction

Colorectal cancer (CRC) is one of the most prevalent malignant digestive tumors, with the third highest incidence rate and the second highest mortality rate worldwide^1^. In the US alone, approximately 149,500 new cases and 52,980 deaths were reported in 2021^2^. The incidence rate of CRC among people younger than 50 years has increased by about 2% annually, while the death rate also increased by approximately 1.3% per year in this age group^3^. Colon adenocarcinoma (COAD) is the most common subtype of colorectal malignant tumor, experiencing a declining 5-year survival rate despite advancements in clinical treatments such as surgery, chemotherapy, and radiotherapy^4,5^. This underscores the urgent need for a deeper understanding of molecular mechanisms and the discovery of novel biomarkers in COAD^6^.

Polyamines, products of a rate-limiting reaction catalyzed by ornithine decarboxylase (ODC), are essential for cellular growth and are pivotal in cancer biology due to their role in cell proliferation and differentiation^7^. Elevated polyamine levels are associated with cancer progression and poor prognosis in multiple cancer types^8^. Ornithine decarboxylase antizyme 2 (OAZ2) plays a critical regulatory role in this context by inhibiting both ODC activity and polyamine uptake, while promoting ODC degradation^9^, thus acting potentially as a tumor suppressor.

OAZ2 is increasingly recognized for its distinct roles in various cancers^10,11^. For instance, Geerts D et al. highlighted that OAZ2 could serve as a significant predictor correlated with the prognosis of neuroblastoma tumors^12^. Li Y et al. demonstrated that overexpression of OAZ2 can rescue the decreased chemosensitivity induced by the deficiency of miR-34a in certain cancer types^13^. Furthermore, Cho LY et al. identified OAZ2 rs1800566 as an important single nucleotide polymorphism (SNP) and found it dramatically decreased in gastric cancer, suggesting a protective role in this context^14^. These findings indicate that while OAZ1 and OAZ2 share functional similarities, OAZ2 exhibits unique potential in cancer prognosis.

Despite its established significance in other cancers, the role of OAZ2 in COAD has been less explored. This presents a unique opportunity to investigate OAZ2 as a novel biomarker in COAD, which could lead to better survival and prognosis rates. Our study aims to dissect the specific role and mechanism of OAZ2 in colon adenocarcinoma, leveraging the capabilities of high-throughput sequencing technology and robust public databases like The Cancer Genome Atlas (TCGA). By doing so, we hope to discover a new potential biomarker that could significantly improve clinical outcomes for COAD patients.

## Methods

### Data acquisition

RNA-seq FPKM data for OAZ2, along with associated clinical information for 39 normal and 398 COAD samples, were sourced from the TCGA Data Portal. Data standardization and analysis were performed using a log2 transformation in R language version 4.0.3^15^, the corroboration information was obtained from Tumor Immune Estimation Resource (TIMER) database (http://timer.cistrome.org/). Overall survival (OS) was evaluated as a prognostic measure. Differentially expressed genes (DEGs) were identified using the ‘limma’ package, applying a threshold of |log2 FC|≥ 1 and an adjusted P-value (FDR) < 0.05^16,17^.

### Gene set enrichment analysis (GSEA)

GSEA, conducted via the Molecular Signatures Database (MSigDB), assessed whether OAZ2 expression could predict significant differences between two COAD sample groups^18,19^. The analysis included over 1000 permutations, identifying significant pathways with a normalized P-value < 0.05 and a normalized enrichment score (NES) > 1.5.

### Survival analysis and ROC analysis

COAD patients were stratified into high and low-risk groups based on median OAZ2 expression. Kaplan–Meier survival analysis and log-rank tests assessed survival disparities^20,21^. A ROC model, developed using the “survivalROC” package, computed the area under the curve (AUC) to evaluate the prognostic sensitivity and specificity of OAZ2 expression^22^.

### Uni- and multivariate cox hazard regression analysis

Analyses were performed to determine if OAZ2 and other clinicopathological parameters (age, gender, race, stage, T, M, N) are independent prognostic indicators for COAD patients, using the corresponding R package^23,24^.

### Nomogram for prognosis prediction

A nomogram was established to predict survival probabilities at 1-, 3-, and 5-year intervals for COAD patients, utilizing the R “rms” package^25,26^. This model illustrated the prognostic impact of various predictors, including demographic and tumor-related factors alongside OAZ2 expression.

### Sangerbox online tool

This tool was employed to explore the relationship between OAZ2 expression and various immune parameters, such as TMB, MSI, neoantigens, and immune cell pathways^27,28^.

### PPI network and HPA database

The STRING website facilitated PPI analysis to identify genes associated with OAZ2^29^. Additionally, the Human Protein Atlas (HPA) database was used to confirm OAZ2 protein expression in COAD via immunohistochemical staining^30^.

### Statistical analysis

Statistical analyses were conducted using R 4.0.3 software. OAZ2 expression differences between normal and tumor tissues were analyzed using the ‘limma’ package and Student’s t-test^31^. The Wilcoxon signed-rank test and logistic regression evaluated associations between OAZ2 expression and clinicopathological variables^32,33^. Kaplan–Meier and ROC curves were generated using the ‘survivalROC’ package^34^. Uni- and multivariate Cox hazard regression analyses identified independent prognostic factors. A nomogram model visualized survival rates using the ‘rms’ package.

### Quantitative real-time PCR analysis (qRT-PCR)

Ten pairs of COAD tissues were collected from the Department of Gastrointestinal Surgery, Affiliated Hospital of Nantong University and qRT-PCR analysis was carried out to reveal the differential expression of OAZ2 and AZIN2 among these tissues. TRIzol reagent (Invitrogen, America) was utilized to extract the total RNA, and Primmer5.0 software was adopted to design the primmer. included OAZ2 (Forward: 5’-ACCTCACATCGTCCACTCCA-3’; Reverse:5’-TTGCTCCCATCAGCTAATAATCC-3’), AZIN2 (Forward: 5′-GCTTAGAGGGAGCCAAAGTG-3′; Reverse: 5′-CTCAGCAAGGATGTCCACAC-3′); and GAPDH (Forward: 5’-GACTTAGTTGCGTTACACCCTTTC-3’; Reverse: 5’-CTGCTGTCACCTTCACCGTTG-3’). Reverse transcription was performed on 2 μg of total RNA using a reverse transcription kit. PCR amplification was conducted under SYBR Green conditions, with the following thermal cycling protocol: an initial denaturation at 95 °C for 2 min, followed by 40 cycles of 95 °C for 15 s, annealing at the corresponding temperature for 15 s, and extension at 72 °C for 30 s. GAPDH was used as an internal reference, and each sample was analyzed in triplicate.

### Western blot analysis

Protein extraction was carried out by lysing collected tissue and cells in Radioimmunoprecipitation Assay (RIPA) buffer. The cell lysates were centrifuged at 12,000 rpm for 10 min at 4 °C to remove cellular debris. The supernatant was collected, and 5X Loading buffer was added. The protein samples were then heated to denature before being loaded onto an SDS-PAGE gel for electrophoresis, facilitating protein separation.

Following electrophoresis, proteins were transferred from the gel to a polyvinylidene difluoride (PVDF) membrane using a wet transfer system. The membrane was then blocked with 5% bovine serum albumin (BSA) in Tris-buffered saline with 0.1% Tween-20 (TBST) for 2 h at room temperature to prevent non-specific binding.

The membrane was incubated overnight at 4 °C with gentle shaking using primary antibodies diluted in TBST. The primary antibodies used were anti-OAZ2 (biorbyt, catalog no. biorbyt), anti-AZIN2 (abcam, ab192771), and anti-GAPDH (Proteintech, 60,004–1-Ig) at a dilution ratio of 1:1000. Post incubation, the membrane was washed three times with TBST for 10 min each to remove any unbound antibodies.

Subsequently, the membrane was incubated with horseradish peroxidase (HRP)-conjugated secondary antibodies diluted 1:1000 in TBST for 1 h at room temperature with gentle shaking. After three washes with TBST for 10 min each, chemiluminescent detection was employed to visualize the protein bands.

GAPDH was used as a loading control to ensure equal protein loading and transfer across the samples. The experiments were performed in triplicates for each sample to ensure reproducibility and reliability of the results.

### Cell culture

Human colon adenocarcinoma cells (RKO) were purchased from the Shanghai Cell Bank of the Chinese Academy of Science (Shanghai, China) and were cultured in Dulbecco’s Modified Eagle Medium (DMEM) supplemented with 10% Fetal Bovine Serum (FBS) and 100 units per milliliter of penicillin–streptomycin. Cells were maintained at 37 degrees Celsius in a humidified atmosphere containing 5% CO2.

### Cell scratch assay

RKO cells were cultured in 6-well plates until they reached approximately 80% confluence. After removing the growth medium, a sterile pipette tip was used to scratch across the cell monolayer, creating a uniform wound. The cells were then washed with phosphate-buffered saline (PBS) to remove detached cells. Fresh medium containing 5% FBS was added to facilitate cell migration into the wound area. Migration was monitored and photographed at 0, 24, and 48 h post-scratch using a microscope equipped with a camera. Wound closure was quantitatively analyzed by measuring the gap in the scratch at each time point.

### Plasmid transfection

RKO cells were plated at 60–70% confluence and transfected with a plasmid encoding the OAZ2 gene using a commercial transfection reagent according to the manufacturer’s instructions. After 4–6 h of incubation with the transfection mixture, the medium was replaced with fresh complete medium. Cells were further cultured for 24–48 h to allow for expression of the transfected gene. Selection with an appropriate antibiotic, as recommended by the plasmid manufacturer, was applied every 48 h to maintain a population of stably transfected cells. These cells were subsequently cryopreserved in liquid nitrogen for future analyses.

### CCK-8 cell proliferation assay

For the cell proliferation assay, 5,000 RKO cells were seeded per well in a 96-well plate and allowed to adhere for 24 h. Subsequently, 10 µl of CCK-8 reagent were added to each well containing 100 µl of growth medium. After incubating for two hours, the absorbance at 450 nm was measured using a plate reader. Cell viability was calculated relative to the control group and expressed as a percentage of the control.

## Results

### Expression levels of OAZ2 in COAD from TCGA, TIMER and HPA database

**Fig. 1:**
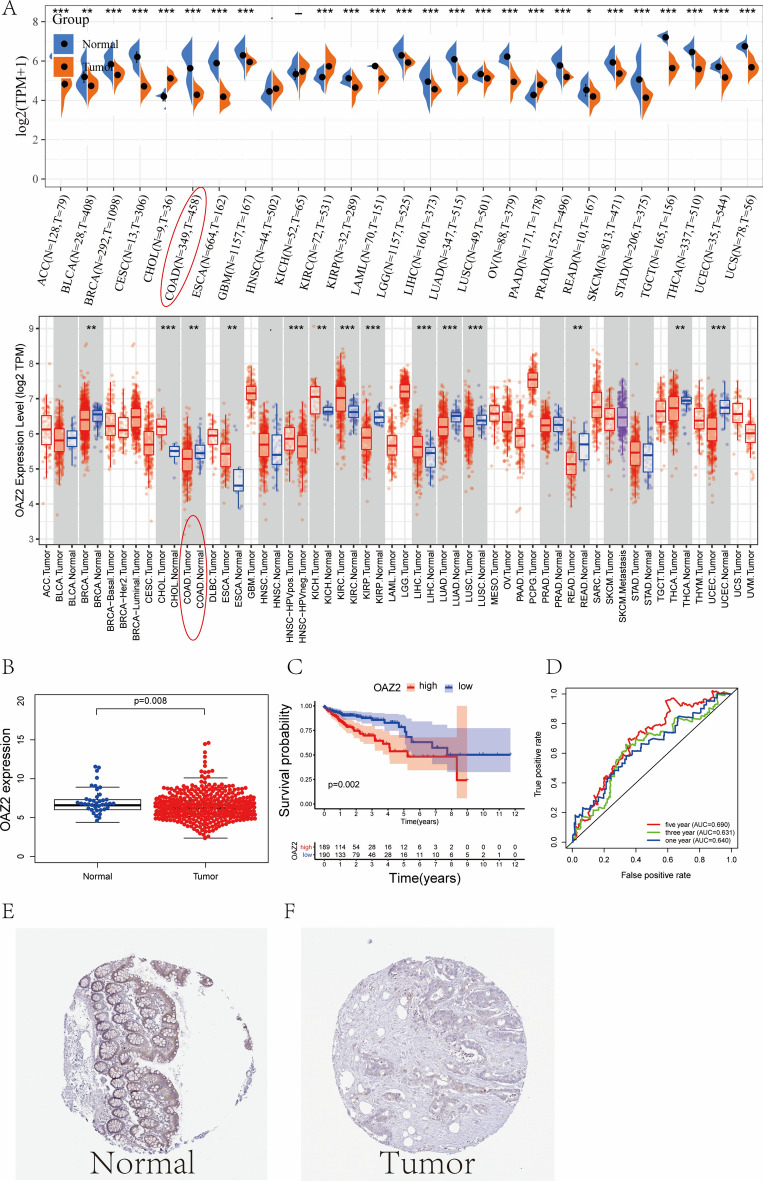
OAZ2 Expression in Colon Adenocarcinoma (COAD). (**A**) OAZ2 expression levels across various cancers based on TCGA data and TIMER database. (**B**) Boxplot showing OAZ2 expression in matched normal and COAD tissues (N = 39; T = 398). (**C**) Kaplan–Meier survival curves for OAZ2 expression in COAD from TCGA. (**D**) Receiver operating characteristic (ROC) curves for 1-, 3-, and 5-year survival probabilities related to OAZ2 expression. (**E–F**) Immunohistochemical staining illustrating OAZ2 protein expression in normal and COAD tissues from the Human Protein Atlas database.

We assessed the mRNA expression levels of OAZ2 across a variety of tumors using the TCGA database. These expression levels exhibited notable variability, and corroborative analysis using the TIMER database confirmed these findings (Fig. 1A). OAZ2 expression was found to be lower in tumor tissues compared to normal tissues (P < 0.05) (Fig. 1B). Kaplan–Meier survival analysis indicated that the OAZ2-low level group had higher overall survival rates than the OAZ2-high level group (P = 0.02) (Fig. 1C). ROC curves for 1-, 3-, and 5-year survival probabilities showed AUCs of 0.640, 0.631, and 0.690, respectively (Fig. 1D). Immunohistochemical staining from the HPA database revealed moderate OAZ2 expression in normal colon tissues but not in COAD tissues (Fig. 1E, F).

### Association between OAZ2 expression and clinicopathologic parameters

**Fig. 2:**
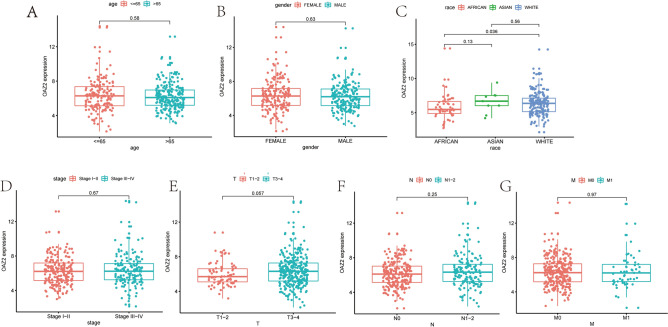
Associations Between OAZ2 Expression and Clinicopathologic Parameters. (**A**) Correlation between OAZ2 expression and age. (**B**) Correlation between OAZ2 expression and gender. (**C**) Correlation between OAZ2 expression and race. (**D**) Correlation between OAZ2 expression and cancer stage. (**E**) Correlation between OAZ2 expression and T stage. (**F**) Correlation between OAZ2 expression and N stage. (**G**) Correlation between OAZ2 expression and M stage.

Logistic regression and the Wilcoxon signed-rank test were utilized to explore the relationship between OAZ2 mRNA expression and seven clinicopathological parameters (age, race, gender, stage, T, M, N). No significant associations were found among these factors (all P > 0.05) (Fig. 2A-D).

### OAZ2 as an independent prognostic factor

**Fig. 3:**
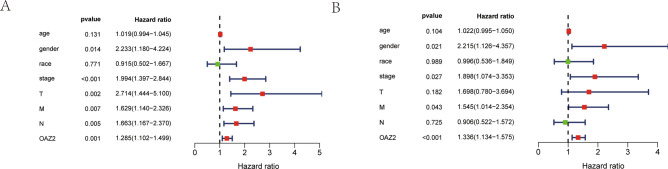
Uni- and Multivariate Cox Regression Analyses. (**A**) Univariate Cox regression analysis of clinicopathologic parameters: age, gender, race, stage, T, M, N, and OAZ2 expression. (**B**) Multivariate Cox regression analysis of the same parameters: age, gender, race, stage, T, M, N, and OAZ2 expression.

Uni- and multivariate Cox regression analyses identified stage, gender, M and OAZ2 expression as independent prognostic factors for COAD patients (all P < 0.05) (Fig. 3A, B; Table 1).

**Table 1:** Univariate and Multivariate Cox Regression Analyses of Clinicopathologic Parameters in COAD from the TCGA Database.

	Univariate analysis				Multivariate analysis			
Characteristics	HR	HR.95 L	HR.95 H	pvalue	HR	HR.95 L	HR.95 H	pvalue
Age	1.02	0.99	1.05	0.13	1.02	1.00	1.05	0.10
Gender	2.23	1.18	4.22	0.01	2.22	1.13	4.36	0.02
Race	0.91	0.50	1.67	0.77	1.00	0.54	1.85	0.99
Stage	1.99	1.40	2.84	0.00	1.90	1.07	3.35	0.03
T	2.71	1.44	5.10	0.00	1.70	0.78	3.69	0.18
M	1.63	1.14	2.33	0.01	1.54	1.01	2.35	0.04
N	1.66	1.17	2.37	0.00	0.91	0.52	1.57	0.72
OAZ2	1.29	1.10	1.50	0.00	1.34	1.13	1.57	0.00

### Establishment of COAD prognostic prediction nomogram

**Fig. 4:**
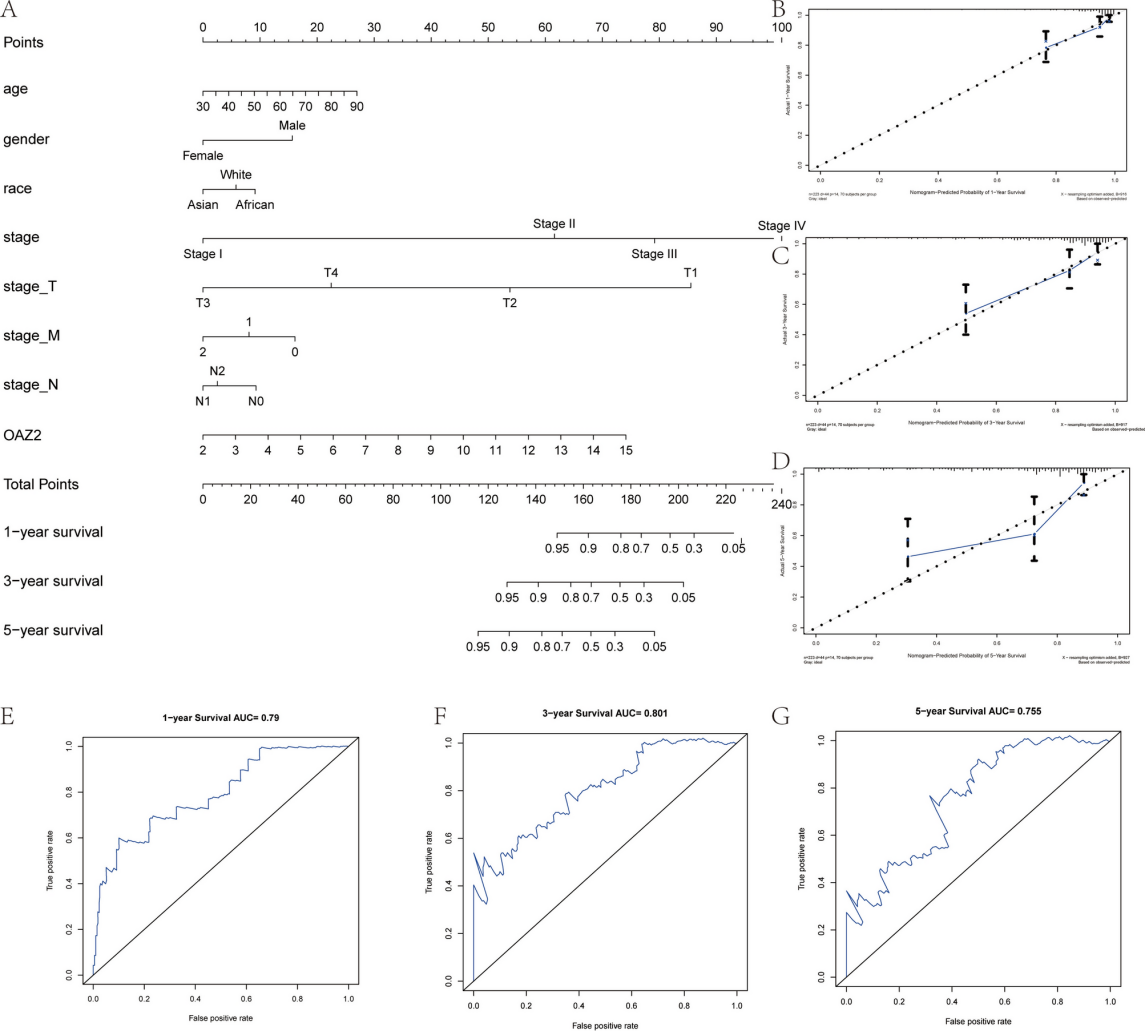
OAZ2-Based Prognostic Nomogram Model and Calibration Curves. (**A**) Prognostic nomogram model incorporating OAZ2 and other clinicopathologic variables. (**B-D**) Calibration curves for 1-, 3-, and 5-year survival probabilities. (**E–G**) AUCs for 1-, 3-, and 5-year survival curves based on TCGA data.

A nomogram model based on clinicopathological parameters and OAZ2 was established to predict 1-, 3-, and 5-year survival rates, achieving a C-index of 0.784 (Fig. 4A). Calibration curves verified the model’s consistency between forecasted and observed outcomes. The AUCs of the survival curves were 0.790, 0.801, and 0.755, indicating medium-level prediction accuracy (Fig. 4B-D). The AUCs of survival curves were 0.790, 0.801, and 0.755, indicating medium-level prediction accuracy (Fig. 4E-G).

### GSEA identified OAZ2-related signaling pathways

**Fig. 5:**
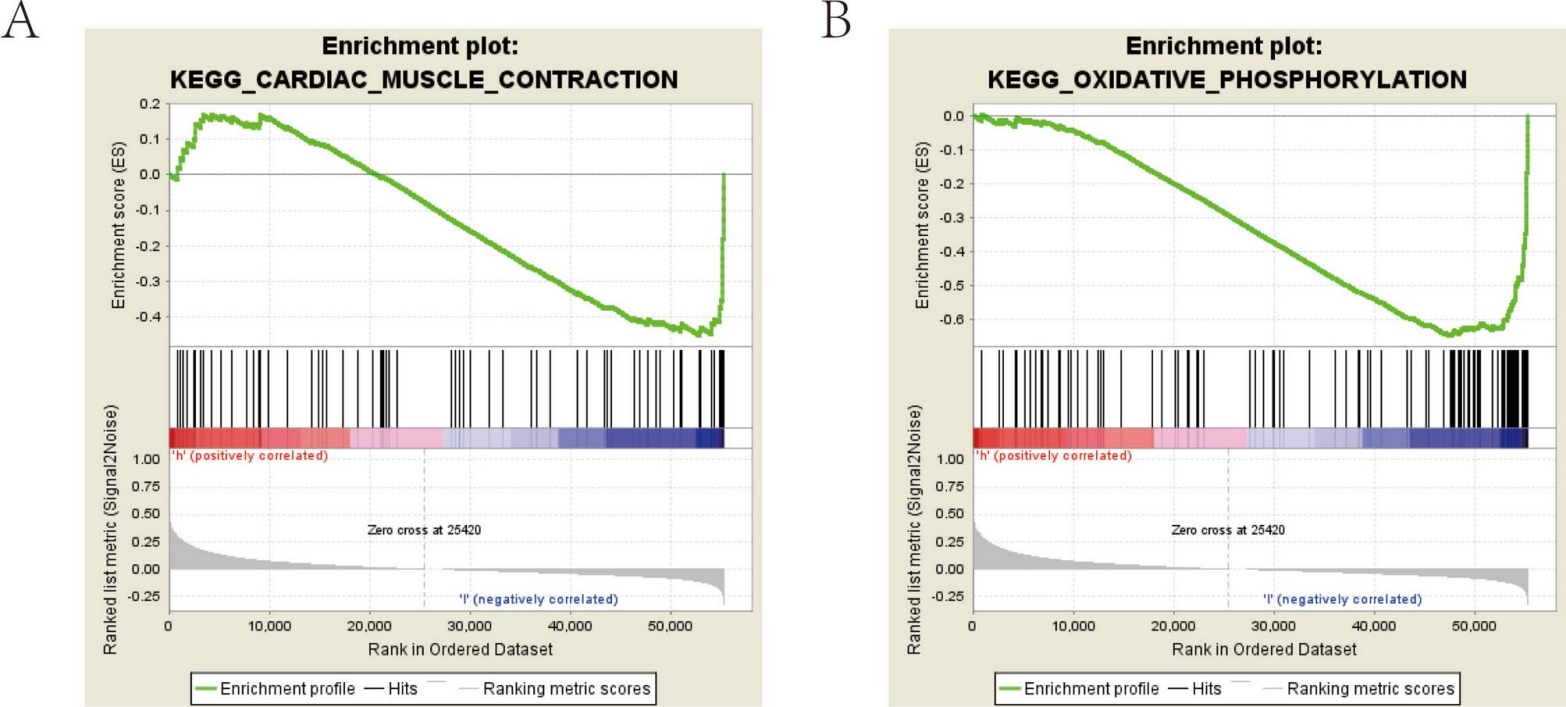
Gene Set Enrichment Analysis Based on OAZ2 in COAD. (**A**) Enriched signaling pathway: cardiac muscle contraction. (**B**) Enriched signaling pathway: oxidative phosphorylation.

GSEA revealed two signaling pathways significantly associated with high OAZ2 expression. The first, cardiac muscle contraction, showed a negative enrichment score (NES) of −1.534 with a normalized p-value of 0.046 and a false discovery rate (FDR) q-value of 0.256. The second pathway, oxidative phosphorylation, exhibited a NES of −1.739, a normalized p-value of 0.030, and an FDR q-value of 0.426. These findings suggest that OAZ2 may play a role in these critical biological processes within COAD (normalized P-value < 0.05, NES > 1.5) (Fig. 5A, B).

### Relationships between OAZ2 and PPI network, MSI, TNB, TMB in COAD

**Fig. 6:**
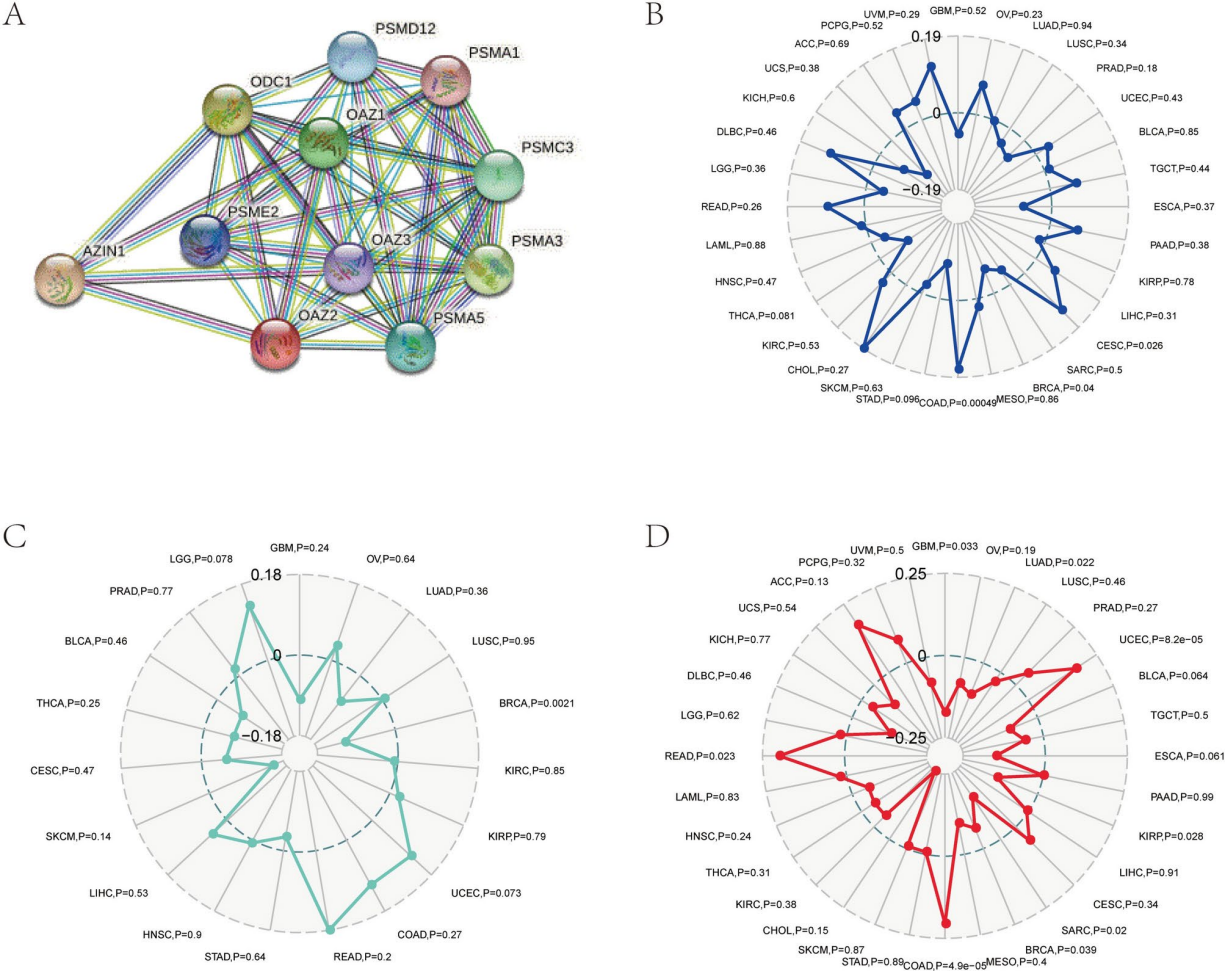
Associations Between OAZ2 and PPI Network, MSI, TNB, TMB in COAD. (**A**) Protein–protein interaction network from TCGA database. (**B**) Association between OAZ2 and MSI. (**C**) Association between OAZ2 and TNB. (**D**) Association between OAZ2 and TMB.

PPI analysis identified 10 genes closely related to OAZ2 expression (Fig. 6A). Pearson’s method highlighted strong associations between OAZ2 expression and MSI (P = 0.00049) and TMB (P = 4.9e-05), but not TNB (P = 0.27) (Fig. 6B-D).

### Tumor immune infiltration and tumor microenvironment

**Fig. 7:**
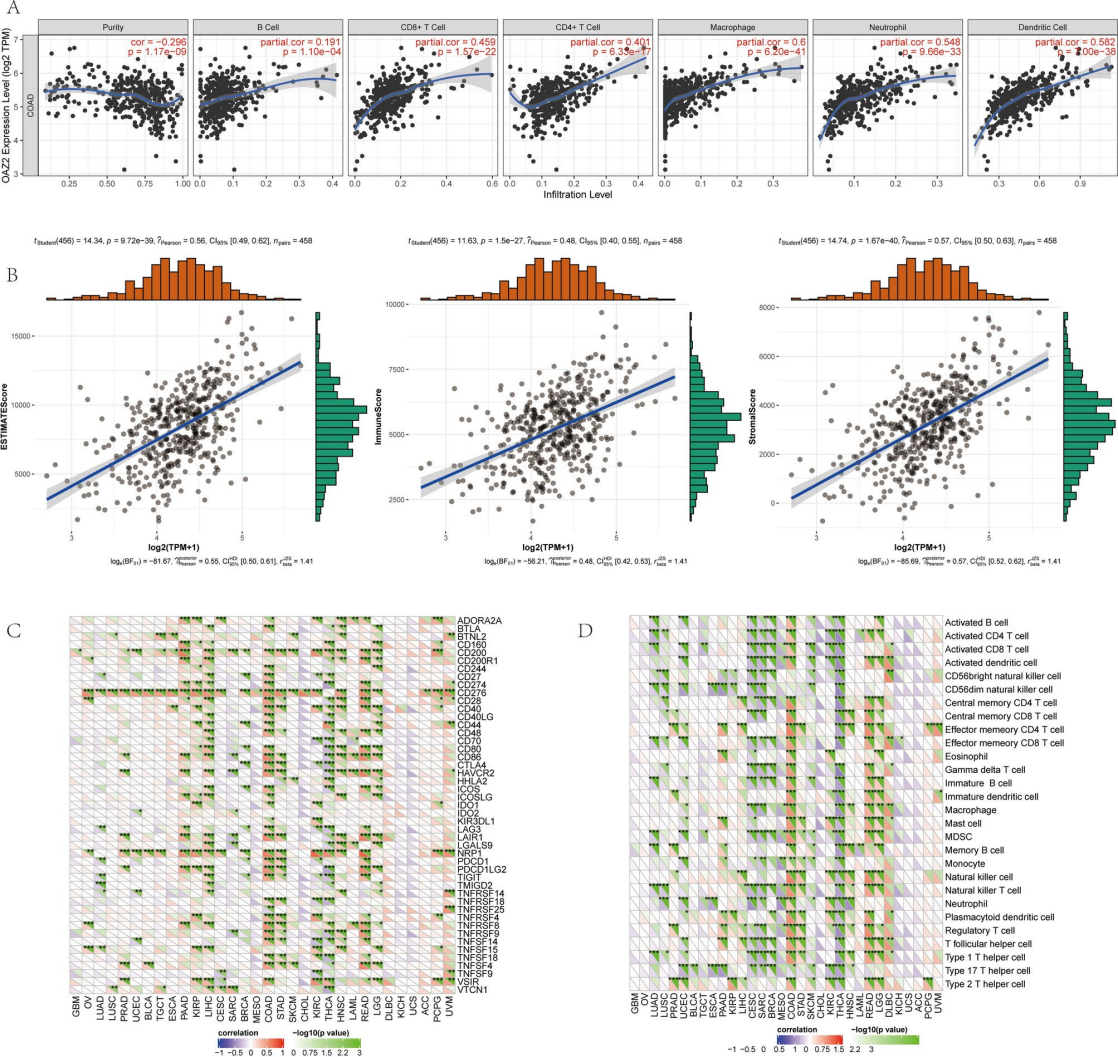
Associations Between OAZ2 Expression and Immune Factors in COAD. (**A**) Association between OAZ2 expression and tumor immune infiltration. (**B**) Association between OAZ2 expression and tumor environment. (**C**) Association between OAZ2 expression and immune checkpoint molecules. (**D**) Association between OAZ2 expression and immune cell pathways.

OAZ2 mRNA expression was strongly associated with several levels of tumor immune infiltration and correlated with ESTIMATEScore, ImmuneScore, and Stromal Score (all P < 0.05) (Fig. 7A, B).

### Correlations between OAZ2 and immune checkpoint molecules, immune cells pathway in COAD

Significant associations were found between OAZ2 expression and various immune checkpoint molecules and immune cell pathways (all P < 0.05) (Fig. 7C, D), emphasizing its potential role in immune regulation within the COAD microenvironment.

### Impact of OAZ2 on proliferation and migration of colorectal cancer cells and its expression analysis in COAD tissues

**Fig. 8:**
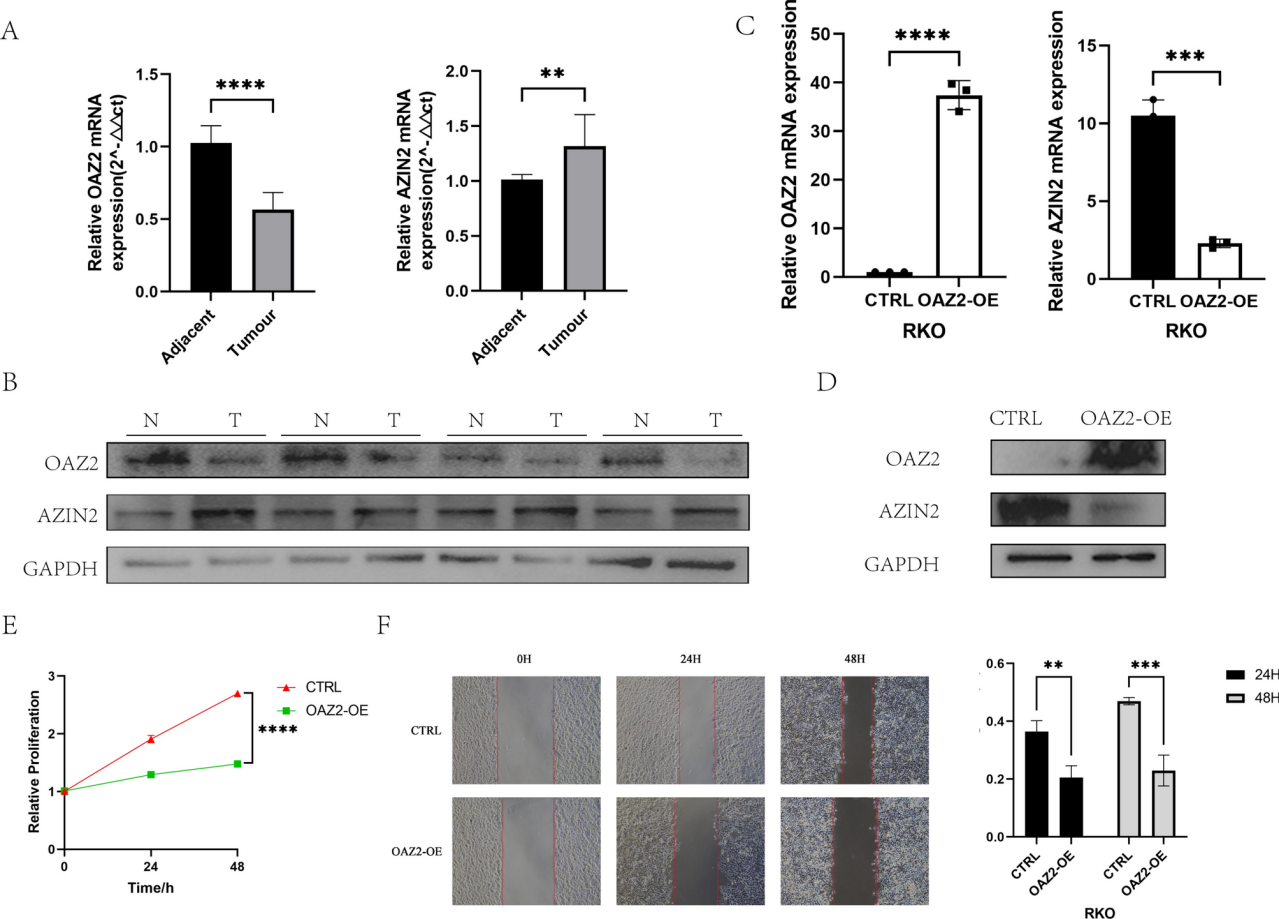
OAZ2 Regulates the Proliferation and Migration of Colorectal Cancer Cells. (**A**) qRT-PCR analysis of OAZ2 and AZIN2 mRNA levels in 10 pairs of clinical tissues. P < 0.01 indicates significant differences. (**B**) Western blot analysis of OAZ2 and AZIN2 protein expression in four randomized pairs of clinical tissues (original blots are presented in Supplementary 1). (**C**) qRT-PCR analysis of mRNA levels of OAZ2 and AZIN2 in ordinary RKO cells and RKO cells overexpressing OAZ2. **P < 0.0001 denotes highly significant differences. (**D**) Western blot analysis comparing OAZ2 and AZIN2 protein levels in regular RKO cells and those overexpressing OAZ2. **P < 0.0001 highlights significant expression changes (original blots are presented in Supplementary 1). (**E**) CCK-8 cell proliferation assay results for RKO cells and those overexpressing OAZ2, with cell activity measured at 24 and 48 h. **P < 0.0001 shows significant differences in proliferation. (**F**) Scratch assay results for RKO cells and those overexpressing OAZ2, photographed at 24 and 48 h. Quantitative analysis indicates significant differences in migration rates, with P < 0.01 and *P < 0.001.

To evaluate the biological role of Ornithine Decarboxylase Antizyme 2 (OAZ2) in colorectal cancer cell dynamics, quantitative real-time PCR (qRT-PCR) analysis revealed that OAZ2 mRNA levels are higher in normal tissues compared to colorectal adenocarcinoma (COAD) tissues **(**Fig. 8A**)**. This differential expression was corroborated at the protein level, where Western blot analysis of tissues randomly selected from the cohort also showed reduced OAZ2 protein expression in cancer tissues compared to adjacent normal tissues **(**Fig. 8B**)**.

Interestingly, the expression of AZIN2, the antagonist of OAZ2, demonstrated a reverse trend both at the mRNA and protein levels, suggesting a competitive interplay between these two molecules. In COAD tissues, AZIN2 mRNA and protein levels were elevated compared to adjacent normal tissues, indicative of a possible compensatory mechanism or competitive inhibition by OAZ2 **(**Fig. 8A** and **Fig. 8B**)**.

To further investigate the effects of OAZ2 on colorectal cancer cell behavior, we transfected RKO cells with an OAZ2 overexpression plasmid. The successful overexpression of OAZ2 was confirmed via both qRT-PCR and Western blot, showing not only increased levels of OAZ2 but also a subsequent suppression of AZIN2 mRNA and protein expressions. This finding supports the hypothesis of a competitive regulatory relationship between OAZ2 and AZIN2 **(**Fig. 8C** and **Fig. 8D**)**.

Functional assays following OAZ2 overexpression revealed significant changes in cell behavior. The CCK-8 proliferation assay showed that RKO cells with overexpressed OAZ2 had a reduced proliferation rate compared to control cells **(**Fig. 8E**)**. Additionally, scratch assays indicated that these cells also displayed impaired migration capabilities, with slower wound closure observed at both 24 h and 48 h post-scratch **(**Fig. 8F**)**.

## Discussion

Ornithine decarboxylase (ODC) plays a crucial role in polyamine biosynthesis, which is essential for cellular proliferation, differentiation, and apoptosis^35^. Excessive accumulation of ODC has been shown to promote tumorigenesis and has been detected in various epithelial cancers, including those of the colon, skin, and prostate^36^. Antizyme, particularly OAZ1, is known to suppress tumor growth by facilitating the degradation of ODC^37^. Similarly, OAZ2 not only inhibits polyamine transport but also acts as a tumor suppressor in specific cancers such as gastric cancer^14^and neuroblastoma^12^. Our results further demonstrate that OAZ2 mRNA and protein levels are significantly reduced in COAD tissues compared to adjacent normal tissues, underscoring its potential tumor suppressive role in colorectal cancer. Additionally, the increased expression of AZIN2, the antagonist of OAZ2, in COAD tissues suggests a competitive interplay that could influence polyamine biosynthesis and tumor dynamics^38,39^. Cho et al. demonstrated that the polymorphism rs7403751 in OAZ2 is associated with a reduced risk of gastric cancer under high daidzein concentrations^14^, while He et al. confirmed that OAZ2 enhances the function of OAZ1 in maintaining follicle homeostasis by inhibiting polyamine biosynthesis^10^. Furthermore, Li et al. highlighted that OAZ2 is a direct downstream target of the tumor-suppressor miR-34a, and its overexpression can mitigate the chemosensitivity impairment caused by miR-34a deficiency in colon cancer^13^.

Despite these findings, research on the prognostic roles of OAZ2 in colorectal cancer (CRC) remains scarce. Our study is pioneering in reporting a correlation between low OAZ2 expression and poor prognosis in colon adenocarcinoma (COAD) patients. We observed that OAZ2 expression is significantly lower in tumor tissues compared to adjacent normal tissues and is closely linked with overall survival (OS). Our systematic analysis of RNA-seq data from the TCGA database for COAD indicates that factors like gender, stage, metastasis (M), and OAZ2 expression can independently serve as prognostic indicators for COAD patients.

Experimental investigations into the biological role of OAZ2 in colorectal cancer cell dynamics revealed that overexpression of OAZ2 in RKO cells significantly reduces their proliferation and impairs migration, as shown by CCK-8 proliferation assays and scratch tests. These findings support the hypothesis that OAZ2 directly influences cell behavior, further validating its role as a tumor suppressor and providing insights into potential therapeutic strategies targeting OAZ2 and its regulatory pathways.

Gene Set Enrichment Analysis (GSEA) identified significant associations between low OAZ2 expression and key signaling pathways such as cardiac muscle contraction and oxidative phosphorylation. The CARDIAC_MUSCLE_CONTRACTION pathway, primarily known for its role in heart function, includes components that influence cellular mechanisms such as calcium signaling, which is also crucial in cancer cell dynamics. This pathway’s involvement in COAD might reflect underlying changes in cellular metabolism and signaling that contribute to tumor progression and patient prognosis. Previous research has established that oxidative phosphorylation (OXPHOS) and glycolysis are crucial for ATP production in tumor cells, enhancing CRC malignancy and drug resistance^40–42^. Our findings confirm the significant role of OXPHOS in colorectal cancer proliferation. Other studies, such as those by Qi et al. and Mahdevar et al., have also linked cellular respiration and the oxidative phosphorylation pathway with cancer progression^43^. Liu et al. demonstrated that the Let-7a microRNA suppresses CRC progression by inhibiting the OXPHOS pathway through SNAP23^44^.

We found no significant correlation between OAZ2 expression and patient age in our COAD cohort. However, age may still influence gene expression patterns indirectly by affecting the tumor microenvironment and immune responses. Prior studies suggest that age-related changes in immunity could impact tumor progression and gene expression^45^. Further investigations are needed to explore how these age-associated factors might interact with OAZ2 expression and influence prognosis in colorectal cancer.

Nomogram models, recognized for their convenience and predictive accuracy, are increasingly used in oncology^46^. Huang et al. developed a nomogram that incorporates seven variables, which was shown to predict overall survival in patients with low-grade endometrial stromal sarcoma^47^. Similarly, Zeng et al. created a model that surpasses existing prognostic tools for breast cancer^48^. In our study, we constructed a nomogram that includes OAZ2 expression and various clinicopathological parameters (age, gender, race, stage, T, M, N). The area under the curve (AUC) values for 1-, 3-, and 5-year survival rates, together with calibration curves, substantiate the model’s predictive reliability.

The competition between AZIN2 and OAZ2, which impacts polyamine levels and influences cellular proliferation and apoptosis, highlights the complex regulation of polyamine metabolism. AZIN2’s role in upregulating ODC activity might indirectly diminish the tumor-suppressive effects of OAZ2 by enhancing polyamine biosynthesis. The implications of AZIN2’s interaction with OAZ2 warrant further investigation to elucidate detailed mechanisms and potential therapeutic targets.

Lastly, tumor mutational burden (TMB) and microsatellite instability (MSI) continue to serve as independent indicators for the efficacy of immune checkpoint inhibitors (ICPIs)^49,50^. Our study reveals a robust correlation between OAZ2 expression and MSI, TMB in COAD patients, indicating potential implications for immunotherapy strategies. These findings underscore the importance of understanding the tumor environment and immune system interactions in predicting treatment responses.

## Conclusions

Our study confirms OAZ2 as a significant prognostic indicator in colon adenocarcinoma (COAD), with a strong association to immune-related processes. We identified two critical signaling pathways through Gene Set Enrichment Analysis (GSEA)—cardiac muscle contraction and oxidative phosphorylation—that are linked to OAZ2 expression and may influence the aggressiveness of COAD.

qRT-PCR confirmed OAZ2’s differential expression in COAD versus normal tissues, supporting its biomarker potential. In vitro studies showed that OAZ2 overexpression significantly inhibits COAD cell migration and proliferation, highlighting its therapeutic value.

The development of a robust nomogram model integrating OAZ2 expression and other clinicopathological factors effectively predicts the prognosis of COAD patients, demonstrating high predictive reliability and accuracy.

Our findings support the potential of OAZ2 as a valuable biomarker and suggest its role as a therapeutic target, especially for modulating immune responses in the tumor microenvironment. Further research is necessary to fully elucidate the mechanisms by which OAZ2 influences COAD and to explore its implications in personalized medicine.

## Supplementary Information

Supplementary Information.

## Data Availability

The datasets generated and analyzed during the current study are available in The Cancer Genome Atlas (TCGA) Data portal (https://portal.gdc.cancer.gov/). All data supporting the findings of this study are included in the article and its supplementary information files. Further inquiries can be directed to the corresponding author.

## Abbreviations

AUC: Area Under the Curve
AZIN2: Antizyme Inhibitor 2
COAD: Colon Adenocarcinoma
CRC: Colorectal Cancer
DEGs: Differentially Expressed Genes
GSEA: Gene Set Enrichment Analysis
MSI: Microsatellite Instability
MSigDB: Molecular Signatures Database
OAZ2: Ornithine Decarboxylase Antizyme 2
ODC: Ornithine Decarboxylase
OS: Overall Survival
OXPHOS: Oxidative Phosphorylation
PPI: Protein–Protein Interaction
ROC: Receiver Operating Characteristic
SNPs: Single Nucleotide Polymorphisms
TCGA: The Cancer Genome Atlas
TIMER: Tumor Immune Estimation Resource
TMB: Tumor Mutational Burden
TNB: Tumor Neoantigen Burden
