# Supplementary figures and images for: Ornithine decarboxylase antizyme 2 (OAZ2) in human colon adenocarcinoma: a potent prognostic factor associated with immunity
Source: Sci Rep. 2025 Mar 3;15:7481. doi: 10.1038/s41598-025-90066-4 (PMC11876682; doi:10.1038/s41598-025-90066-4)

The complete WB images of Figure 8.B


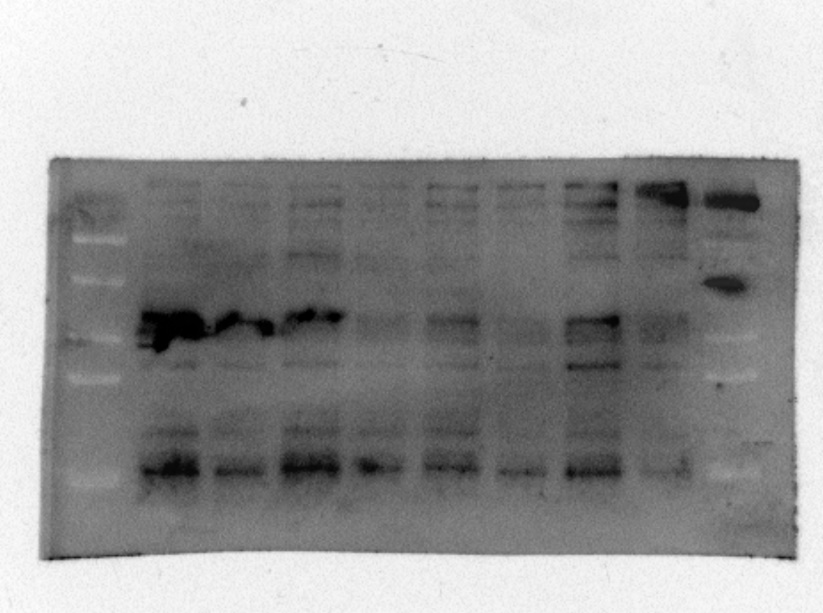

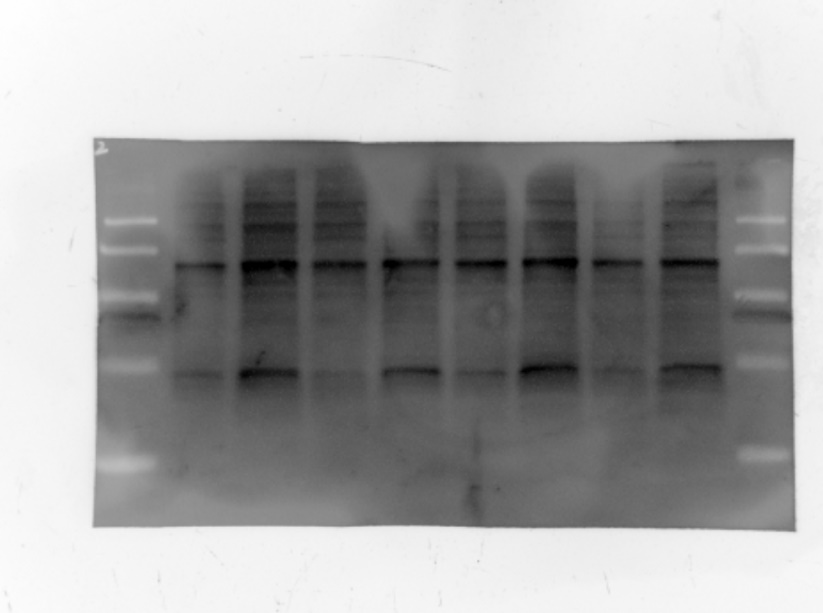

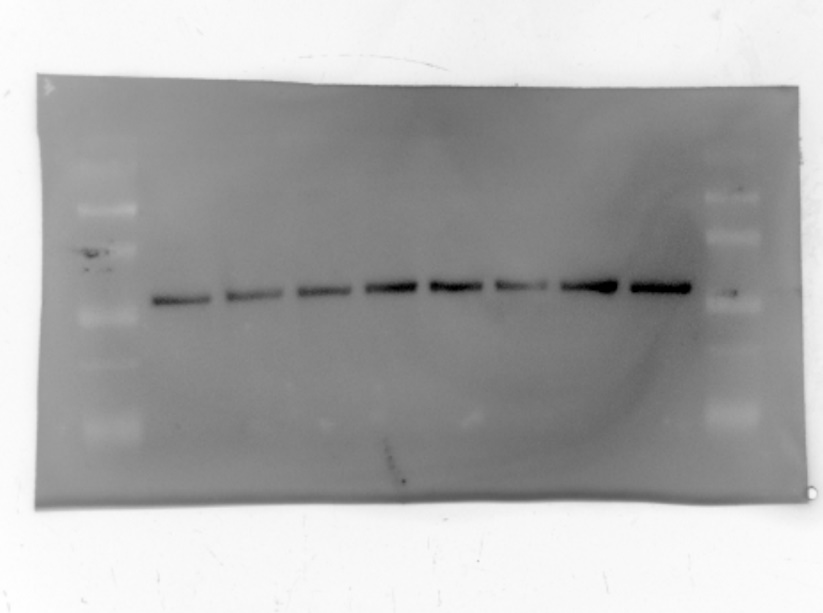


The complete WB images of Figure 8.D


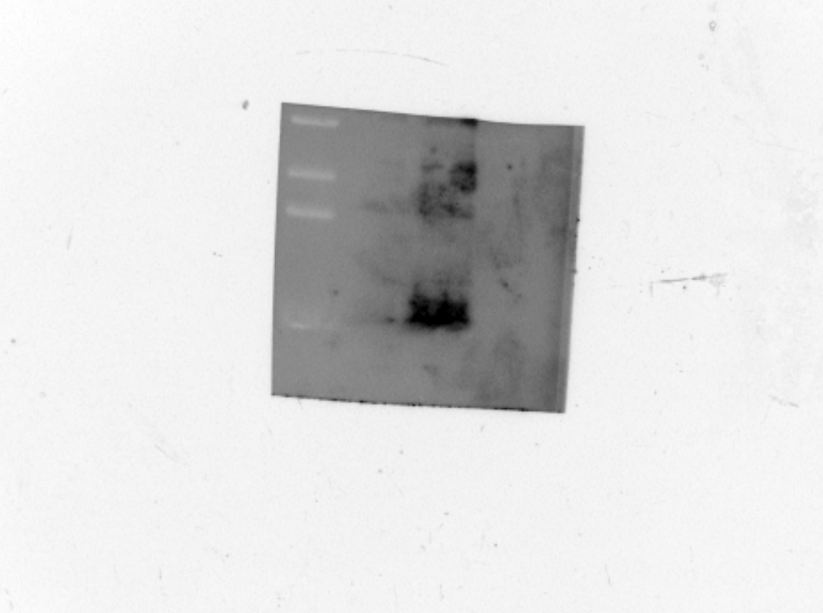

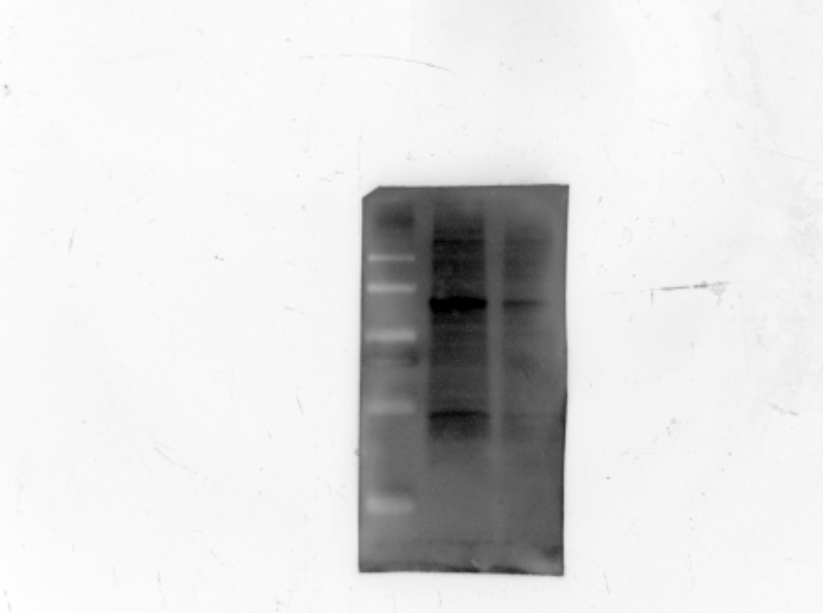

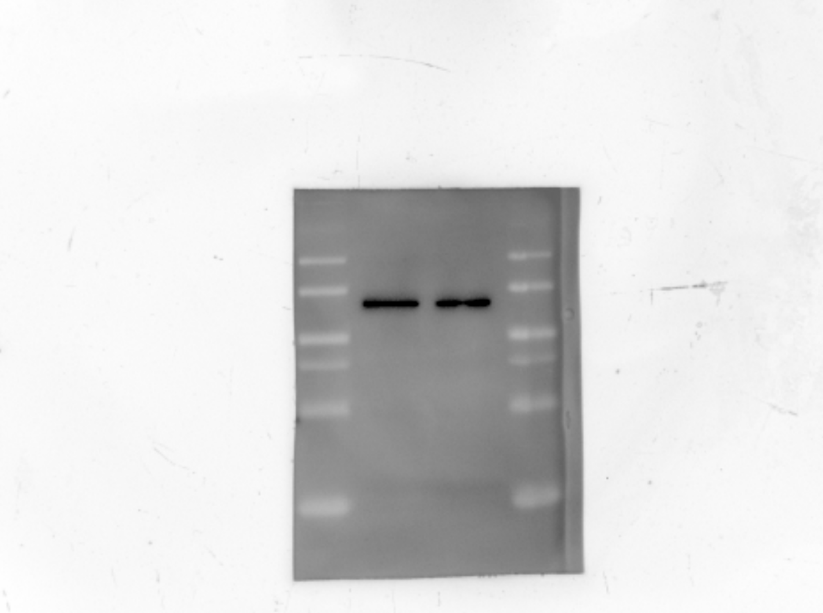

Supplement: Supplementary file 1 — Supplementary Information. [file 41598_2025_90066_MOESM1_ESM.docx]
